# Significance of the Lung Immune Prognostic Index for Assessment of the Reliability of the Clinical Treatment Outcome for Advanced Non-Small-Cell Lung Cancer in Patients with COVID-19 Infection

**DOI:** 10.3390/jcm11226695

**Published:** 2022-11-11

**Authors:** Kristina Krpina, Martina Mavrinac, Miroslav Samarzija, Ena Tolic, Dora Darapi, Lara Baticic

**Affiliations:** 1 Clinic for Respiratory Diseases Jordanovac, University Hospital Centre Zagreb, 10000 Zagreb, Croatia; 2 Department of Medical Informatics, Faculty of Medicine, University of Rijeka, 51000 Rijeka, Croatia; 3 Faculty of Medicine, University of Zagreb, 10000 Zagreb, Croatia; 4 Department of Medical Chemistry, Biochemistry and Clinical Chemistry, Faculty of Medicine, University of Rijeka, 51000 Rijeka, Croatia

**Keywords:** chemotherapy, immunotherapy, lung immune prognostic index, non-small-cell lung cancer, COVID-19

## Abstract

**Introduction:** Lung cancer is one of the most diagnosed malignancies with increasing incidence worldwide. Immunotherapy is the main oncological treatment for advanced non-small cell lung cancer (NSCLC), for which the discovery of new efficient biomarkers is crucial. Scientific evidence points to the importance of the Lung Immune Prognostic Index (LIPI), but its predictive significance is unclear. **Aim:** The aim of this study was to investigate the clinical significance and predictive role of LIPI in patients with advanced NSCLC and PD-L1 mutation who are eligible for immunotherapy in combination with chemotherapy. In addition, to our knowledge, this is the first time that the association between COVID-19 infection and the course and outcome of oncologic treatment of NSCLC has been investigated. **Patients and Methods:** Patients were divided into four study groups according to strictly defined clinical parameters, therapeutic approach, and COVID-19 infection. LIPI was determined and its predictive power was evaluated in all studied groups, as well as overall survival (OS), progression-free survival (PFS), and disease control rate (DCR). **Results:** This study confirmed the understudied and uncertain predictive power and clinical relevance of LIPI as a biomarker in patients with advanced NSCLC. Patients infected with COVID-19 had a higher survival rate than uninfected patients despite the therapeutic approach, which may be attributed to their hospitalization and intensive medical management during the pandemic. **Conclusions:** Findings obtained in this study may help to determine treatment options according to the clinical condition of the patient by using LIPI values as a non-invasive, readily available and economically acceptable predictive biomarker in lung oncology.

## 1. Introduction

Lung cancer (LC) is among the most frequently diagnosed cancer types and the leading cause of cancer-related deaths with cumulatively increasing incidence worldwide. The treatment of lung cancer requires a multidisciplinary approach due to the complexity of the disease, therapeutic possibilities of personalized medicine, and the knowledge of genetic changes in carcinogenesis, which are essential in selecting an adequate therapy [1]. Although cytotoxic chemotherapy has long been the mainstay of systemic treatment for lung cancer, it has given way to the precision of targeted therapy and the power of immunotherapy. Interestingly, those two strategies are somewhat at odds with each other in the management of non-small-cell lung cancer (NSCLC): tumors without actionable targets will not respond to targeted therapy [2,3], and those with certain targets are unlikely to respond to immunotherapy [4].

Research conducted in recent years has expanded knowledge about the role of the immune system in tumor development and progression. Immunotherapy that uses immune checkpoint inhibitors has provided one of the most important breakthroughs in the management of solid tumors, including lung cancer, and has shown promising results in numerous clinical trials [5]. Immune checkpoint inhibitors (ICIs), particularly inhibitors of the PD-1 axis, have altered the therapeutic landscape of NSCLC [6,7]. The most intensely studied checkpoint pathways in NSCLC are the PD-1 pathways, which incorporate receptor PD-1 and its reciprocal ligands (programmed death-ligand 1 and 2, PD-L1 and PD-L2, respectively), and the cytotoxic T-lymphocyte antigen-4 (CTLA-4) pathways. Monoclonal antibodies targeting PD-1 (nivolumab, pembrolizumab), CTLA-4 (ipilimumab), or PD-L1 (durvalumab, atezolizumab, avelumab) have brought significant improvements in the progression-free survival (PFS) and overall survival (OS) compared with second-line chemotherapy in late-phase clinical trials and have been rapidly established as standard management of advanced stage NSCLC [8,9,10,11]. 

Biomarkers, a crucial segment in immuno-oncology, are still under intensive scientific research, in addition to the PD-L1 mutation and the tumor mutation burden (TMB). However, due to the heterogeneity of PD-L1 mutations and TMBs and their mismatch in scoring systems, they are not suitable for use as predictive markers in immuno-oncology [12,13,14,15]. Scientific efforts are focused on finding a reliable marker that would have both prognostic and predictive value, with economic acceptability and ease of determination, where “liquid” markers are a promising alternative to tissue markers [16,17]. The prognostic and predictive value of several inflammatory-related markers, such as neutrophil–lymphocyte ratio (NLR) and derived neutrophil–lymphocyte ratio (dNLR), has been shown to play a major role in the destruction of cancer cells. Mezquita et al. in 2018 were the first to propose the lung immune prognostic index (LIPI), which integrates baseline dNLR and LDH values before treatment—two risk factors for poor prognosis easily available in clinical practice [18]. Furthermore, Ruiz–Bañobre et al. confirmed the usefulness of LIPI as a prognostic marker in 2019 [19]. In the same year, Sorich et al. [20] described in a cohort of 1489 patients that LIPI is a reliable prognostic marker that can identify groups of patients treated with atezolizumab with significantly different survival outcomes and responses. However, although the prognostic value of LIPI has been confirmed, its predictive value is still uncertain [16,21].

The emerging pandemic caused by COVID-19, due to the specificity of the respiratory tract infection it causes, significantly endangers lung cancer patients. Respiratory disorders, immune response, lymphopenia, and cytokine cascades caused by this virus play a fundamental role in the development and severity of symptoms [22]. One of the most important diagnostic features in individuals with COVID-19 is lymphocyte depletion. Due to the stimulation of interferon-γ (INF-γ) production by neutrophils and monocytes, which are abundant in the peripheral blood of people with COVID-19, the expression of inhibitory immune checkpoints, including PD-1, PD-L1, and CTLA4, on the surface of T lymphocytes is enhanced. According to previous research, the PD-1/PD-L1 pathway has been shown to represent an escape mechanism for certain pathogens, and the use of anti-PD-L1 could increase the clearance of some viruses [22,23,24,25]. Based on the positive effect that ICI has on the reactivation of T lymphocytes against cancer cells, as well as cells infected with the virus, it can be assumed that the use of ICI is unlikely to pose a risk to cancer patients during this pandemic and can be proposed in the therapy of cancer patients infected with COVID-19. Therefore, we hypothesized that ICIs might play a protective role against COVID-19 infection. 

This research is further based on the hypothesis that LIPI represents an effective predictive marker when determining treatment outcomes with a certain therapeutic approach to patients with metastatic NSCLC and positive PD-L1 mutation. The main goal of this research was to evaluate the clinical significance and predictive value of LIPI as a marker in the prognosis of the treatment outcome in patients with metastatic NSCLC and positive PD-L1 mutation treated with the first-line oncology treatment (immunotherapy or combination of chemotherapy and immunotherapy). Furthermore, we aimed to investigate, to our knowledge for the first time, the role of LIPI in patients with metastatic NSCLC and a positive PD-L1 mutation who had suffered from COVID-19 infection and have continued the treatment with immunotherapy or a combination of chemotherapy and immunotherapy. 

## 2. Materials and Methods

### 2.1. Patients

The research was conducted at the Clinic for Pulmonary Diseases Jordanovac, Clinical Hospital Center Rebro, Zagreb, Croatia. The Clinical Hospital Center is fully equipped and has its own laboratory for the analysis of the variables that were necessary for the conduction of this research. The study was conducted from January 2019 to December 2021, which includes a two-year follow-up of patients included in this research. The research was designed as a retrospective case-control study, in which previously collected data and materials from defined groups of patients were analyzed. 

**Figure 1:**
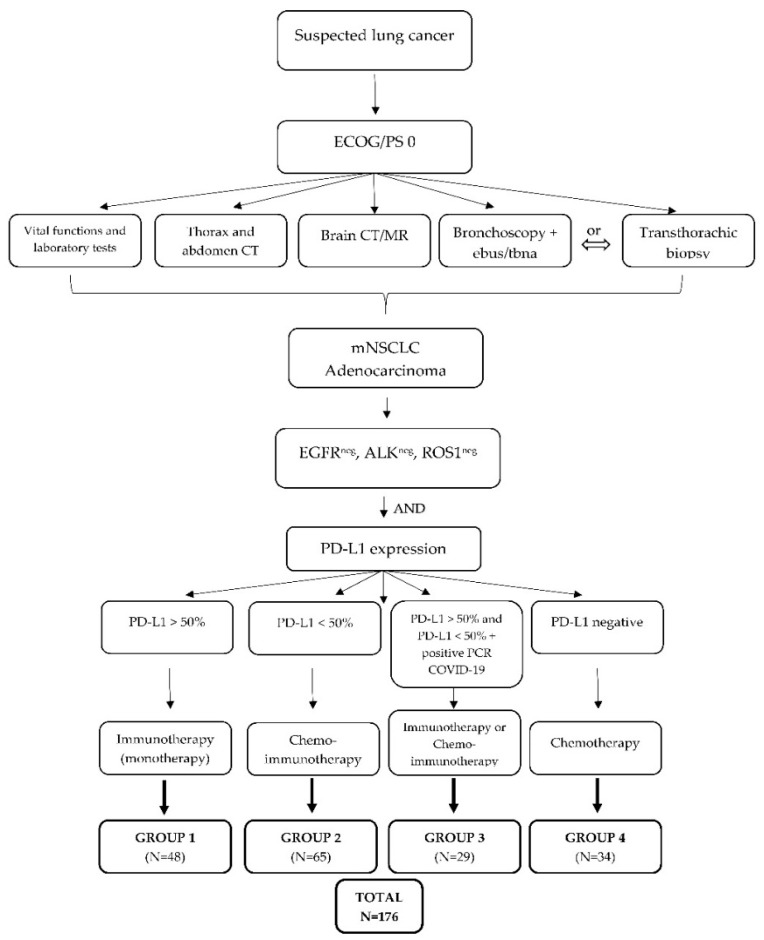
Study algorithm and classification of groups of patients. ECOG/PS, ECOG performance status; mNSLC, metastatic non-small-cell lung carcinoma; EGFR, epidermal growth factor receptor; ALK, anaplastic lymphoma kinase; PD-L1, programmed death-ligand 1.

The research included four groups of patients that were divided according to previously clearly defined parameters and strict inclusion and exclusion criteria, as presented in Figure 1. All patients were diagnosed with metastatic NSCLC with good performance status (ECOG 0–1) and divided according to PD-L1 expression into group 1: patients treated with immunotherapy as monotherapy (PD-L1 > 50%), group 2: patients treated with chemotherapy and immunotherapy combination (PD-L1 < 50%), group 3: patients who recovered from COVID-19 infection after being diagnosed with metastatic NSCLC and treated with immunotherapy as monotherapy or a combination of chemotherapy and immunotherapy, and group 4: patients treated with chemotherapy (the control group). Patients with poor general and clinical condition (ECOG 2–4) and patients who had more than two lines of systemic treatment and whose life expectancy was less than 3 months were excluded from this study as per exclusion criteria. All patients involved in the research were familiar with the purpose and methodology of the research and consented to participate in the research with written informed consent, as well as consent to physical examination and laboratory and diagnostic processing. Medical documentation available from the information system of the Clinic for Pulmonary Diseases Jordanovac was used for the collection of patients’ demographic and clinical data.

### 2.2. Methods

All patients underwent a routine clinical examination that included general history-taking, physical status, assessment of ECOG status, and complete diagnostic workup that included examination of vital functions, biochemical laboratory parameters, and diagnostic radiological processing (X-rays of the heart and lungs and computed tomography analyses). Depending on the clinical and diagnostic findings, further interventional treatment of patients was performed, which included bronchoscopy with tissue collection for pathohistological and cytological analysis and transthoracic biopsy under computed tomography control, i.e., the location of the tumor or metastatic changes.

The expression of PD-L1 was determined by analyzing samples of isolated tumor tissues processed by the standard method of embedding in paraffin blocks and then cut into 4-µm-thick tissue cuts for further immunohistochemical analysis. A standard immunohistochemical method was used in the analysis of PD-L1 expression in lung tumor cells, which included the use of anti-PD-L1 rabbit primary monoclonal antibody (Roche diagnostics, Basel, Switzerland, SP 263) at a dilution of 1:100. After the standard procedure of secondary antibody binding at a dilution of 1:500 and visualization of PD-L1 expression according to the manufacturer’s protocol, an experienced histopathologist analyzed the samples in a double-blinded assay. The expression of at least 1% was considered positive.

Radiological evaluations were performed every 8 to 12 weeks according to valid criteria—MSCT of the thorax and abdomen (assessment of response to oncological treatment) in both the examined groups and the chemotherapy cohort. The radiological response to therapy was evaluated as follows: stable disease (the treatment was continued), partial response (the treatment was also continued), and disease progression (the treatment was discontinued).

The lung immune prognostic index (LIPI), as one of the “liquid” markers currently in the research phase in immuno-oncology and the era of precision medicine, was developed based on two elements: dNLR greater than 3 (according to the limit value from the largest published studies on cancer patients treated with checkpoint inhibitors) and LDH greater than ULN (upper limit of reference values, defined according to the limit of each clinical center). Therefore, depending on the calculated LIPI value (number of factors), three groups were formed: good—0 factors; intermediate—1 factor; and poor—2 factors. 

### 2.3. Statistical Analysis 

Categorical data collected during the research are presented in absolute and relative values, whereas numerical data are presented in appropriate measures of centrality and dispersion, depending on the sample size and the normality of the data distribution. Comparisons of categorical data were calculated with the Chi-square test or Fisher’s exact test. The comparison of numerical variables and calculation of differences between groups were calculated with T-tests and ANOVA, i.e., their non-parametric versions, depending on the characteristics of the data. Survival analysis was calculated by using the Kaplan–Mayer method and the log-rank test. 

Disease control rate (DCR) was defined as a complete plus partial response plus stable disease, and overall response rate as a complete plus partial response. Overall survival (OS) was calculated from the date of the first immunotherapy administration until death due to any cause. Progression-free survival (PFS) was calculated from the date of first immunotherapy administration until disease progression or death due to any cause.

Statistical analyzes were performed with MedCalc version 19.1.7 (MedCalc Software, MariaKerke, Belgium) and Statistica version 13.5.0.17 (StatSoft Inc., Tulsa, OK, USA). All *p* values lower than 0.05 were considered statistically significant.

## 3. Results

The baseline characteristics of the 176 patients included in this research are summarized in Table 1. Patients were divided into four previously defined groups and included 117 (66.5%) male and 59 (33.5%) female patients. The median age was 63 years (range 29–91 years); all of them had good performance status (ECOG 0-1) and were diagnosed with metastatic NSCLC with different PD-L1 expression (according to group classification). Regular radiological findings were performed and noted. According to our results, a complete response was observed in 27.3% of patients (N = 48), a partial response in 36.9% of patients (N = 65), a stable disease in 16.5% of patients (N = 29), and disease progression in 19.3% of patients (N = 34).

**Figure 2:**
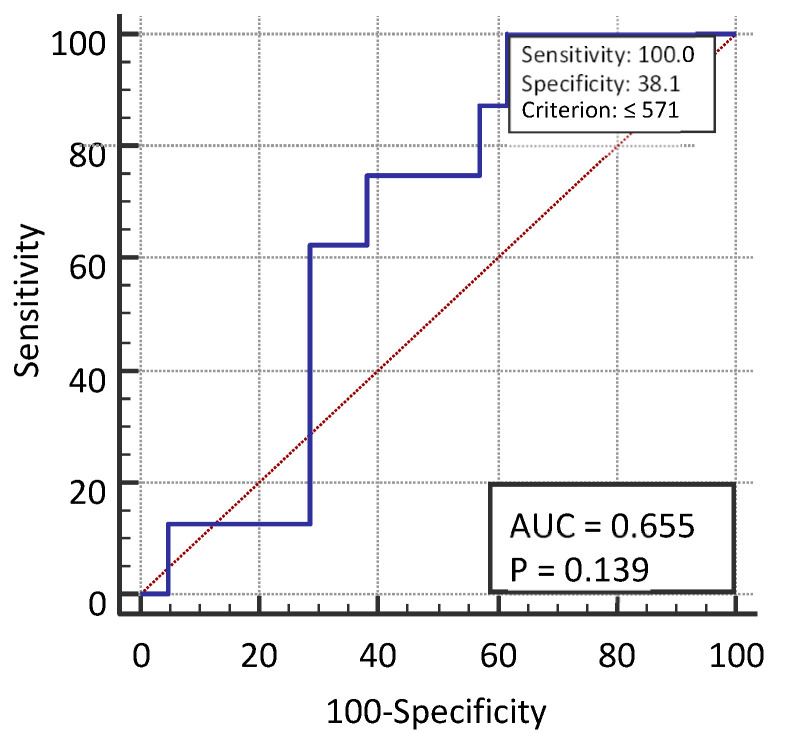
Median follow-up of patients from diagnosis to disease progression.

The median follow-up of patients was 9 months (95% CI, 7–12 months). The median overall survival (OS) was 9.5 months (95% CI, 5.3–12.3 months), and the median progression-free survival (PFS) was 6.9 months (95% CI, 5.9–8.6 months). Statistical analysis of the obtained data on the survival of patients within each group revealed that subjects from group 4 had a statistically significantly higher death rate compared with other groups included in this study (*p* = 0.041) (Figure 2).

### 3.1. Overall Survival of Patients

**Figure 3:**
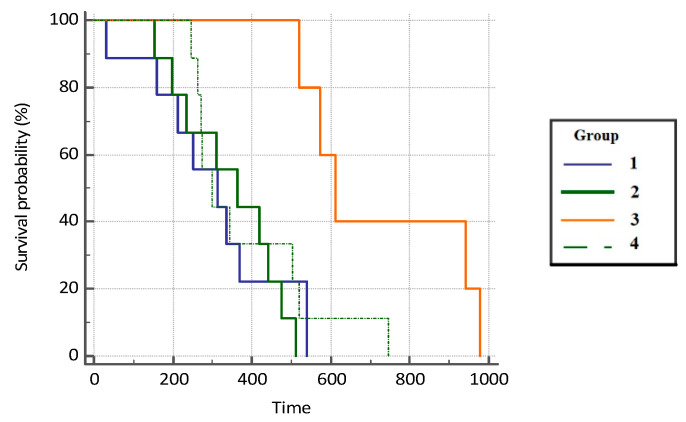
Overall survival probability (OS, %) in analyzed groups of patients.

The difference in OS in patients was statistically significant between the groups included in this study (*p* = 0.039). Patients with COVID-19 infection had a 3.86 times higher survival rate compared with the subjects on immunotherapy (95% CI, 1.46–10.24), 3.98 (HR) times higher survival compared with the patients on chemotherapy who did not suffer from COVID-19 (95% CI, 1.49–10.65), and 3.01 (HR) times higher survival compared with the control group (1.22–7.38) (Figure 3 and Table 2). 

### 3.2. Progression-Free Survival (PFS) of Patients

**Figure 4:**
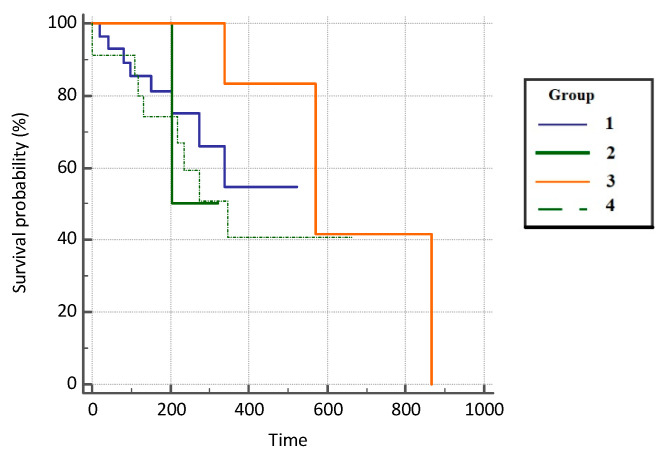
Progression-free survival (PFS) in analyzed groups of patients.

Patients with COVID-19 infection had a 1.91 hazard ratio (HR) times higher progression-free survival rate compared with the subjects on immunotherapy (95% CI, 0.66–5.55), 3.01 HR times higher survival compared with the patients on chemotherapy who did not suffer from COVID-19 infection (95% CI, 0.23–39.25), and 2.73 times higher survival compared with the control group (0.89–8.41). However, the difference in mortality was not statistically significant between the groups (*p* = 0.451) due to the insufficient number of respondents. Results are presented in Figure 4 and Table 3.

### 3.3. Disease Control Rate (DCR)

**Figure 5:**
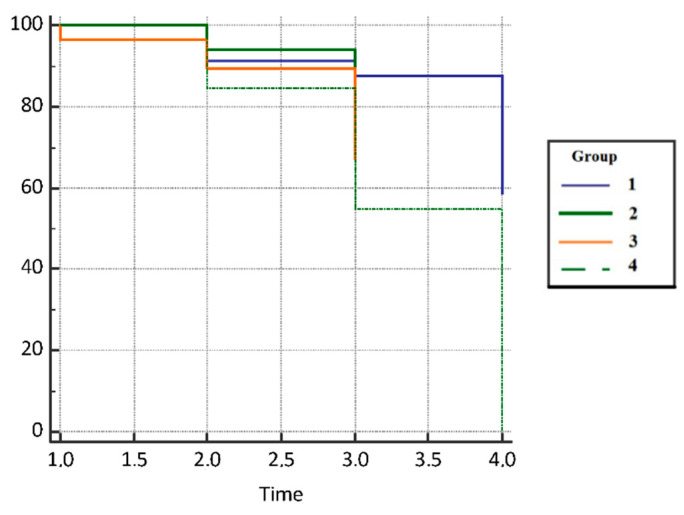
Disease control rate (DCR) in analyzed groups of patients.

Disease control rate (DCR) was defined as a complete plus partial response plus stable disease, and an overall response rate as complete plus partial response. Frequencies for all studied groups are summarized in Table 4. Patients from the control group had 2.80 HR times lower survival compared with the subjects on immunotherapy (95% CI, 1.11–7.05), 2.56 HR times lower survival compared with the patients on chemotherapy who did not suffer from COVID-19 infection (95% CI, 1.00–6.55), and 1.49 HR times higher survival compared with the control group (0.43–5.17), as presented in Table 5. The difference in mortality was statistically significant between the groups *p* = 0.020. Results are presented in Figure 5.

### 3.4. Lung Immune Prognostic Index (LIPI)

The lung immune prognostic index (LIPI) was calculated for all groups of patients included in this study. Baseline dNLR greater than 3 and LDH greater than ULN were independently combined for the LIPI calculation. The median dNLR ratio was calculated in 76 patients (43%), and the median LDH was calculated in 167 patients (95%). The obtained LIPI values (good—0 factors, intermediate—1 factor, poor—2 factors) for individual groups of patients are shown in Table 6. LIPI value 0 or good LIPI consisted of 78 patients (44%), LIPI value 1 or intermediate LIPI group included 81 patients (46%), and LIPI value 2 or poor LIPI group included 17 patients (10% of the total number of patients). 

According to the statistical analysis of the research results from the examined groups, the LIPI value in patients with lung cancer treated with immunotherapy as monotherapy and patients treated with chemotherapy and immunotherapy combination showed no significant difference. The largest number of patients was in the intermediate LIPI group (group 1), 29 patients (60%) from group 1, and 32 patients (49%) from group 2. However, in the group of patients who recovered from COVID-19 infection (group 3), the largest number was in the good LIPI group (group 0), 17 patients (59%) compared with 12 patients (41%) in the intermediate LIPI group (group 1), and group 2 (poor LIPI) contained no patients. Furthermore, in the control group, the largest number of patients was in the good LIPI group (group 0). 

### 3.5. Clinical Relevance and Predictivity of the Lung Immune Prognostic Index

In all groups of our study, which included 176 patients treated with first-line immunotherapy, median OS and PFS were 9.5 and 6.9 months, respectively, which is consistent with previous reports of patients with advanced NSCLC treated with ICI. According to our results, 10% of patients with poor LIPI had a higher probability of disease progression and had shorter PFS (median value 5.0 months) and OS (median value 8.4 months) than patients with intermediate or good LIPI value.

By analyzing the obtained results, it was determined that the association between LIPI values and OS in the total number of patients was not statistically significant (r = 0.274, *p* = 0.129), as well as the association between LIPI and PFS (r = 0.0400, *p* = 0.7574) and the association between LIPI and DCR (r = 0.0483, *p* = 0.5245). Additionally, we analyzed the correlation of LIPI values and PD-L1 expression in the total sample of subjects. The research results showed there was no statistically significant correlation between LIPI values and PD-L1 expression (PD-L1 negative, greater, or less than 50%, depending on the group of subjects) with overall survival and survival without disease progression (*p* = 0.9164). 

In the chemotherapy cohort, 29 patients with advanced NSCLC were included and had a median follow-up of 6.6 months (95% CI, 0–12.5 months). Baseline characteristics are summarized in Table 1. Patients received a median of one chemotherapy line (range 1–3), which was the first-line treatment for all of them (100%). Median PFS and OS were 8.4 (95% CI, 0–11.6) and 5.0 (95% CI, 1–8.9) months, respectively. 

Median dNLR and LDH amounted to 3.02 (range 0.78–43.11) and 209 IU/L (range 177–232 IU/L), respectively. No correlation was observed in the control cohort between dNLR or LDH and OS or PFS. Among the 29 patients evaluable for LIPI, 22 patients (65%) had a good LIPI score, 8 (24%) intermediate, and 4 (11%) poor scores. No correlation was found between LIPI, OS, PFS, and DCR (all *p* > 0.05).

## 4. Discussion

Lung cancer is one of the leading public health problems worldwide with a low five-year survival rate [3,14]. In the last ten years, the overall approach to lung cancer diagnostic processing, including treatment, has changed and improved with new knowledge and therapeutical possibilities. A multidisciplinary approach is essential due to the complexity of the disease and the therapeutic possibilities of personalized medicine, which is part of modern oncology. Knowledge regarding genetic changes in carcinogenesis is essential when choosing an adequate therapy. Research conducted in recent years has expanded knowledge about the role of the immune system in the development and progression of tumors [12,13,14]. The goal of tumor immunotherapy is to strengthen the host’s weak immune response or to apply one of the forms of passive immunity, for example, by using antibodies. In 2013, Chen et al. [12] were the first to describe the cancer immunity cycle and the mechanism of action of this form of passive immunity by using immune checkpoint inhibitors (including PD-L1), which is still under intensive scientific research [13,14,15,16].

Biomarkers, as an important component of immuno-oncology, are still in the research phase, but the latest scientific findings suggest “liquid” markers, one of which is LIPI, represent a promising alternative to tissue markers. Our main aim in this study was to evaluate the clinical significance and the predictive role of LIPI in patients with advanced NSCLC. Results of our study revealed that LIPI values in our examined groups, in the largest number of patients, a total of 81 (46%), belong to the intermediate LIPI (group 1), whereas 78 patients (44%) belong to the good LIPI (group 0), and only 17 patients (10%) belong to the poor LIPI (group 2). Comparing our results with the study of Mezquita et al. [18], the largest number of patients in group 1 (intermediate LIPI) is also 62 (49%). Similar findings were obtained in the study by Ruiz–Bañobre et al. [19] (N = 63 patients; 33.5%). However, in the research by Huang et al. [26] and Ortega–Franco et al. [27], the largest number of patients was in group 0 (good LIPI)—61 patients (67%) and 54 patients (47.8%). The reason for the above is that for the last two to three years, there has been an exceptional development of targeted diagnostic processing, which is faster, more reliable, and more accurate, and there is no need for retesting. Different screening programs have been developed, resulting in excellent patient responsiveness. All of the above leads to faster detection of lung cancer, unfortunately still as a metastatic disease, but in a clinically good general condition of the patient, which, according to the above analysis, indicates that patients were diagnosed when uncontrolled systemic inflammation had not yet developed. Likewise, this includes a higher value of LDH activity, leading to a better LIPI value.

According to the statistical analysis of our research results, comparing the examined groups, LIPI in group 1 and group 2 showed no significant difference. The largest number of patients was in the intermediate LIPI group—29 patients (60%) vs. 32 patients (49%). However, in group 3, the highest number of patients was in the good LIPI group (17 patients (59%) vs. 12 patients (41%) in the intermediate LIPI group), whereas the poor LIPI group did not have a single patient. In addition, in the control group (group 4), the largest number of patients belongs to the good LIPI group. All of the above indicates that patients, although in the metastatic stage of the disease, started the treatment in good clinical condition and without developing systemic inflammation of the tumor. Our analysis of patients who received ICI or combination treatment is consistent with previously published reports by other research groups [22,23,26,28]. The published results of Mezquita et al. [14] describe that from the total number of patients, 49% were in the intermediate LIPI group. However, we see that our group 3 correlates with more recent papers and meta-analyses: Ortega–Franco [27] and Huang [26] reported 47.8% and 67% of patients in the good LIPI group, whereas Ruiz–Bañobre reported 41% [19]. For the first time, to our knowledge, our research included patients who recovered from COVID-19 infection and received ICI, which represents a new scientific contribution in this context. Because there are still no available or published results analyzing LIPI values in patients who have recovered from COVID-19 infection and received ICI, we cannot discuss our results until there are available results from other researchers.

According to the goals of this research, we calculated the correlations of LIPI with other defined parameters—OS, PFS, and DCR in examined groups of patients. In our total population of 176 patients treated with first-line immunotherapy, median OS and PFS were 9.5 and 6.9 months, respectively, which is consistent with previous reports on patients with advanced NSCLC treated with ICI [18,20,27]. According to our results, 10% of patients with poor LIPI had a higher probability of disease progression as the best response to therapy and had shorter PFS (median 5.0 months) and OS (median 8.4 months) than those with intermediate or good LIPI value.

Additionally, it was determined that the association between LIPI values and OS in the total number of patients is not statistically significant (r = 0.274, *p* = 0.129), as well as the association between LIPI and PFS (r = 0.0400, *p* = 0.7574) and the association between LIPI and DCR (r = 0.0483, *p* = 0.5245). Additionally, we analyzed the correlation of LIPI values and PD-L1 expression in the total sample of subjects. The results of our research showed that there was no statistically significant correlation between LIPI values and PD-L1 expression (PD-L1 negative, greater, or less than 50%, depending on the group of subjects) with overall survival and survival without disease progression (*p* = 0.9164).

Analyzing examined groups and correlating each group, patients from group 3 had the best OS with a median value of 24.9 months (749 days), whereas for patients in group 1 and group 2, it amounted to 10.4 and 14.5 months, respectively. As expected, patients from the control group (group 4) had the worst OS rate, which amounted to only 8.4 months (253 days; 95% CI 0–349). Additionally, the results of our study showed that patients with COVID-19 infection had a 3.45 times higher survival compared with the subjects on immunotherapy and chemotherapy who did not suffer from COVID-19 (HR = 3.45; 95% CI, 1.04–11.46). Adequate COVID-19 vaccination status was associated with a significantly reduced risk of severe disease or mortality: HR = 0.20 (95% CI, 0.17–0.22). Patients included in the study were all vaccinated and were only 20% more likely to get severe COVID-19 disease or die. Likewise, our results show that patients who received combination treatment with ICI and chemotherapy (group 2) compared with the patients who received mono-immunotherapy (group 1) had a longer OS by 4.5 months, indicating a more effective combination treatment.

The mentioned analysis further indicates that patients who recovered from COVID-19 infection during treatment with mono-immunotherapy or combination treatment did not have complications as a result of infection with the virus. Furthermore, there were no restrictions or interruptions in further oncological treatment, but two years of ICI treatment was carried out in all patients. According to the conducted research, we can conclude that patients are either in active follow-up or active further oncological treatment. 

The results of our research, for the first time according to our knowledge and available literature, confirm that a higher value of LIPI (especially in patients who recovered from COVID-19 infection) represents a prognostic and predictive factor for OS in patients treated with ICI, as well as for patients who received chemotherapy. Comparing our results regarding OS (9.5 months vs. 10 months) from the first published paper by Mezquita et al. [18], we observed a slight difference in OS. The difference was not statistically significant between good LIPI and poor LIPI (r = 0.274, *p* = 0.129). According to the latest available literature, the results of the work by Ruiz–Bañobre et al. [19] show a median OS of 12.9 months for patients in the intermediate LIPI group. For the same LIPI group, the results of the study by Ortega–Franco et al. [27] indicate a median OS of 11.8 months, whereas Huang et al. [26] indicate a median OS of 26.2 months. This indicates that with the development of modern diagnostic processing that is available to most hospital centers, together with the earlier detection of lung cancer, and thus with the faster start of oncological treatment, we are monitoring better OS results. In the Republic of Croatia, unfortunately, we still have a long period until the start of oncological treatment—more than two months, which also affects OS results.

Additionally, in our study groups, the best PFS results were again found in group 3 with a median PFS of 11.7 months (353 days), which indicates that COVID-19 infection had no impact on PFS in our patients. Moreover, for the mentioned examined group, the largest number of patients (59%) was in the good LIPI group, and it can therefore be assumed that lower LIPI (good or intermediate) is associated with a better PFS and thus the effectiveness of the immunotherapy itself. In group 1 and group 2, the PFS was 6.6 months (198 days) and 8.7 months (262 days), respectively, which is in line with previously published results [18,20,26,27]. The above indicates that combination treatment with ICI and chemotherapy is more effective than ICI monotherapy. On the other hand, the expected PFS in the control group (group 4) was only 5 months (151 days).

Overall, all patients in the intermediate LIPI group developed disease progression before 14 months, group 1 in 13.5 months and group 2 in 10.6 months. In group 3, the progression developed only after 23.2 months, and in the control group, after 6.6 months, which was expected. In addition, the results of the control group suggest that patients started treatment soon after the diagnosis of advanced NSCLC (with negative PD-L1), with a span of 14 days, with a good LIPI, but due to the poor efficacy of the chemotherapy, they had a rapid progression and started the second-line oncological treatment within 6 months.

So far, there are no published results on the association between LIPI and ICI-treated patients recovered from COVID-19 infection. However, these results are directly related to the limited number of patients in the group 3; therefore, further studies with a larger number of patients are needed to confirm our results. In addition, the results of our study with the group 1 suggested that a poor LIPI score was associated with a worse prognosis in patients with NSCLC. Statistical analysis of the obtained data on the survival of patients within each group revealed that subjects from group 4 had a statistically significantly higher death rate than other groups (*p* = 0.041).

The results of our study confirm previously published analyses in mixed cohorts of patients treated with PD-L1 or anti-PD-L1 inhibitors. Regarding LIPI, the number of relevant studies examining the prognostic value of this factor has increased since the pioneering study by Mezquita et al. [18]. Numerous studies have investigated the prognostic value of LIPI in NSCLC patients receiving immunotherapy or chemotherapy [18,19,20,26,27]. Ruiz–Bañobre et al. also confirmed the importance of LIPI in the prognosis and prediction of disease control in patients with advanced NSCLC treated with ICIs [19]. In more recent works, Ortega–Franco et al. indicate that LIPI is a prognostic factor, but that further analysis is needed to determine its predictive value [27], which was the aim of our study. Different authors investigated the association between LIPI and lung cancer survival outcomes among patients with different types of pathology and under different treatment regimens. However, the prognostic value of LIPI in NSCLC patients remains a divisive issue. In a Japanese study published by Minami et al. [28], it was reported that LIPI is a valuable prognostic factor only for specific subgroups of NSCLC patients treated with a tyrosine kinase inhibitor (EGFR, epidermal growth factor receptor). Statistical analysis showed they had a higher OS, PFS, and disease control rate. They also underwent two years of ICI treatment; all patients were either under active follow-up or in active oncological treatment. All of the above indicates that LIPI is a predictive factor; that is, it indicates what the outcome of immunotherapy treatment could be. Due to the fact that our study was conducted on a smaller number of patients, the hypothesis also requires a prospective analysis based on a larger number of patients.

Furthermore, the new pandemic caused by COVID-19, due to the specificity of the respiratory tract infection it causes, significantly endangers patients with lung cancer. Based on the positive effect that ICIs have on the reactivation of T lymphocytes against cancer cells, as well as virus-infected cells, it is concluded that the use of ICIs is unlikely to pose a risk to cancer patients during this pandemic, and that they can be proposed in the therapy for cancer patients who are infected with COVID-19 [29].

To our knowledge, no relevant clinical studies have been published so far on the impact of LIPI in patients treated with ICI who had recovered from COVID-19 infection. The research phase is still ongoing, and the results are still expected in the coming period. Due to the lack of data on the impact of COVID-19 infection, our research is the first to have investigated the association of the infection with the course and outcome of oncological treatment of lung cancer. Group 3 in this study included patients from the first and second wave of the COVID-19 pandemic treated at the Clinic for Respiratory Diseases Jordanovac, Zagreb. Considering that this was a worldwide pandemic, the entire medical community learned about the virus on a daily basis. It not only affected the entire population, but also had an impact on our cancer patients and the course of their treatment. All of our patients were treated with mono-immunotherapy or a combination of chemotherapy and immunotherapy and recovered from COVID-19 infection. The median number of days from diagnosis to disease progression was 696 days (1 year and 11 months) compared with the group 1 (435 days; 1 year and 2 months) and group 2 (300 days; less than a year), indicating that COVID-19 infections had no impact on the underlying disease and the further course of treatment. On the contrary, patients were treated longer without developing side effects (grade 3 or 4) that would require treatment discontinuation or the development of radiological or clinical progression that would require treatment discontinuation.

The statistical analysis of the results of overall survival or deterioration without disease progression showed that patients with COVID-19 infection had a 3.45 times higher survival compared with the subjects on immunotherapy and chemotherapy who did not suffer from COVID-19 (HR = 3.45; 95% CI, 1.04–11.46). Likewise, they had 1.91 times higher survival compared with the subjects on immunotherapy (95% CI, 0.66–5.55), 3.01 times higher survival compared with the patients on chemotherapy who did not suffer from COVID-19 infection (95% CI, 0, 23–39.25), and 2.73 times higher survival compared with the control group (0.89–8.41), although the difference in mortality was not statistically significant between the groups (*p* = 0.451) due to an insufficient number of subjects. The difference in overall survival is statistically significant between groups (*p* = 0.039). Patients with COVID-19 infection had a 3.86 times higher survival rate compared with the subjects on immunotherapy (95% CI, 1.46–10.24), 3.98 times higher survival compared with the patients on chemotherapy who were infected with COVID-19 (95% CI, 1.49–10.65), and 3.01 times higher survival compared with the control group (1.22–7.38). Based on the above results, we can conclude that COVID-19 infection had no impact on the survival of ICI-treated patients, but those patients had better overall survival and progression-free survival as well as a better disease control rate. The above can be attributed to more dedicated medical care and hospitalization of patients with COVID-19 infection.

To conclude, in recent years, important improvements in survival have been achieved through earlier detection, improved surgical and radiation techniques, and a dramatic paradigm shift in systemic therapy. Nevertheless, new biomarkers are essential in lung cancer research to predict and improve therapeutic outcome. To our knowledge, according to the published research and available literature, this is the first study to evaluate LIPI in patients treated with a combination of ICI and chemotherapy and in patients receiving the aforementioned therapy who have recovered from COVID-19 infection. Analysis of the obtained results shows that LIPI was not associated with outcomes in patients treated with chemotherapy (predictive factor), which raises the hypothesis that LIPI is a predictive factor in patients treated with ICI. Our results show that these patients had a good LIPI. This study confirms the understudied and uncertain predictive power and clinical relevance of LIPI as a biomarker in patients with lung cancer. These results may provide new insights in determining treatment options depending on the clinical status of the patient by using LIPI values as a noninvasive, readily available, and economically acceptable predictive biomarker in lung oncology.

## Figures and Tables

**Table 1:** Demographic, anamnestic, and clinical characteristics of patients included in the study (expressed as frequencies and median values, 95% CI).

Group	Group 1 N = 48 N (%)	Group 2 N = 65 N (%)	Group 3 N = 29 N (%)	Group 4 N = 34 N (%)	Total N = 176 N (%)
Sex (M/F)	31 (65) 17 (35)	45 (69) 20 (31)	15 (52) 14 (48)	26 (76) 8 (24)	117 (66) 59 (34)
Age at diagnosis (median years, range)	62.5 (29–76)	63 (34–78)	65 (42–91)	64.5 (47–77)	63 (29–91)
CRP (median, 95% CI)	9 (3.85–13.9)	9.8 (5.38–17.18)	149 (112–205)	7.9 (0.4–45.47)	10.3 (7.64–14.09)
Days from diagnosis to progression (median, 95% CI)	435 (344–559)	320 (260–343)	696 (634–983)	200 (0–376)	338 (294–365)
dNLR (N/%)	28 (37)	32 (42)	6 (8)	10 (13)	76 (43)
Immunotherapy PD-L1	48 (27)	65 (37)	29 (16)	34 (19)	176 (100)
0	0	0	0	34 (100)	34 (19)
1	48 (100)	0	9 (31)	0	57 (32)
2	0	65 (100)	20 (69)	0	85 (48)
LIPI	48 (27)	65 (37)	29 (16)	34 (19)	176 (100)
0	12 (25)	27 (42)	17 (59)	22 (65)	78 (44)
1	29 (60)	32 (49)	12 (41)	8 (24)	81 (46)
2	7 (15)	6 (9)	0	4 (11)	17 (10)
LDH (IU/L)	48 (29)	65 (39)	29 (17)	25 (15)	167 (95)
Median days (95% CI)	198 (170–221)	177 (162–187)	168 (150–213)	209 (177–232)	182 (174–192)
Overall survival (OS)	9 (30)	1 (3)	2 (6)	20 (63)	32 (18)
Median days (95% CI)	312 (167–515)	437	749	253 (0–349)	285 (160–370)
Progression-free survival	28 (45)	2 (3)	9 (14)	23 (37)	62 (35)
Median days (95% CI)	198 (155–264)	262	353 (200–866)	151 (38–268)	209 (178–260)
Time from diagnosis to first cycle (days)	47 (64)	2 (3)	10 (14)	14 (19)	73 (41)
Median days (95% CI)	218 (139–346)	31	515 (26–420)	45 (37–69)	133 (76–208)
Time from first to last cycle (days)	47 (50)	1 (1)	16 (17)	30 (32)	94 (53)
Median days (95% CI)	269 (208–335)	511	536 (321–607)	163 (116–265)	268 (213–330)
Total number of doses	48 (27)	65 (37)	29 (16)	34 (19)	176 (100)
Median of doses (95% CI)	10 (8–14)	4 (4–5)	21 (13–24)	45 (38–69)	6 (6–8)

dNLR, derived neutrophil–lymphocyte ratio; PD-L1, programmed death-ligand 1; LIPI, lung immune prognostic index LIPI; LDH, Lactate dehydrogenase.

**Table 2:** Hazard ratios (HR) with 95% confidence interval for OS.

Groups	1	2	3	4
Group 1	-	0.7279 (0.08275–6.4034)	0.2901 (0.08725–0.9646)	0.6680 (0.2497–1.7869)
Group 2	1.3737 (0.1562–12.0842)	-	0.3985 (0.04337–3.6617)	0.9176 (0.1115–7.5540)
Group 3	3.4470 (1.0367–11.4608)	2.5092 (0.2731–23.0548)	-	2.3025 (0.7844–6.7584)
Group 4	1.4971 (0.5596–4.0049)	1.0898 (0.1324–8.9715)	0.4343 (0.1480–1.2748)	-

**Table 3:** Hazard ratios (HR) with 95% confidence interval for PFS.

Groups	1	2	3	4
Group 1	-	1.5692 (0.1247–19.7430)	0.5215 (0.1801–1.5107)	1.4285 (0.5068–4.0264)
Group 2	0.6373 (0.05065–8.0180)	-	0.3324 (0.02548–4.3360)	0.9103 (0.07056–11.7444)
Group 3	1.9174 (0.6620–5.5540)	3.0088 (0.2306–39.2531)	-	2.7390 (0.8920–8.4108)
Group 4	0.7000 (0.2484–1.9732)	1.0985 (0.08515–14.1720)	0.3651 (0.1189–1.1211)	-

**Table 4:** Disease control rate (DCR) by analyzed groups of patients.

	Disease Control Rate				N (%)
Group	1 (CR)	2 (PR)	3 (SD)	4 (PD)	
Group 1	00.0% RT	2143.7% RT	1531.2% RT	1225.0% RT	48 (27.3%)
	0.0% CT	25.6% CT	19.2% CT	92.3% CT	
	0.0% GT	11.9% GT	8.5% GT	6.8% GT	
Group 2	00.0% RT	3046.2% RT	3553.8% RT	00.0% RT	65 (36.9%)
	0.0% CT	36.6% CT	44.9% CT	0.0% CT	
	0.0% GT	17.0% GT	19.9% GT	0.0% GT	
Group 3	13.4% RT	1965.5% RT	931.0% RT	00.0% RT	29 (16.5%)
	33.3% CT	23.2% CT	11.5% CT	0.0% CT	
	0.6% GT	10.8% GT	5.1% GT	0.0% GT	
Group 4	25.9% RT	1235.3% RT	1955.9% RT	12.9% RT	34 (19.3%)
	66.7% CT	14.6% CT	24.4% CT	7.7% CT	
	1.1% GT	6.8% GT	10.8% GT	0.6% GT	
TOTAL	3	82	78	13	176
	−1.7%	−46.6%	−44.3%	−7.4%	

CR, complete response; PR, partial response; SD, stable disease; PD, progression of the disease.

**Table 5:** Hazard ratios (HR) with a 95% confidence interval.

Groups	1	2	3	4
Group 1	-	1.09 (0.50–2.39)	1.88 (0.61–5.80)	2.80 (1.11–7.05)
Group 2	0.91 (0.42–1.99)	-	1.72 (0.55–5.37)	2.56 (1.00–6.55)
Group 3	0.53 (0.17–1.65)	0.58 (0.19–1.82)	-	1.49 (0.43–5.17)
Group 4	0.36 (0.14–0.90)	0.39 (0.15–0.99)	0.67 (0.19–2.33)	-

**Table 6:** Calculated LIPI values in analyzed groups of patients.

Lung Immune Prognostic Index (LIPI)	Group 1	Group 2	Group 3	Group 4	Total
	N (%)				
Group 0 (good)	12 (25%)	27 (42%)	17 (59%)	22 (65%)	78 (44%)
Group 1 (intermediate)	29 (60%)	32 (49%)	12 (41%)	8 (24%)	81 (46%)
Group 2 (poor)	7 (15%)	6 (9%)	0	4 (11%)	17 (10%)
Total	48 (27%)	65 (37%)	29 (16%)	34 (19%)	176 (100%)

## Data Availability

Not applicable.
